# Annexin A2 regulates *Mycoplasma bovis* adhesion and invasion to embryo bovine lung cells affecting molecular expression essential to inflammatory response

**DOI:** 10.3389/fimmu.2022.974006

**Published:** 2022-09-08

**Authors:** Hui Zhang, Doukun Lu, Yiqiu Zhang, Gang Zhao, Abdul Raheem, Yingyu Chen, Xi Chen, Changmin Hu, Huanchun Chen, Liguo Yang, Aizhen Guo

**Affiliations:** ^1^ State Key Laboratory of Agricultural Microbiology, Huazhong Agricultural University, Wuhan, China; ^2^ Hubei Hongshan Laboratory, College of Veterinary Medicine, Huazhong Agricultural University, Wuhan, China; ^3^ College of Animal Husbandry and Veterinary Medicine, Southwest Minzu University, Chengdu, China; ^4^ College of Animal Science and Technology, Huazhong Agricultural University, Wuhan, China; ^5^ Key Laboratory of Development of Veterinary Diagnostic Products, Ministry of Agriculture and Rural Affairs, Huazhong Agricultural University, Wuhan, China; ^6^ Hubei International Scientific and Technological Cooperation Base of Veterinary Epidemiology, Huazhong Agricultural University, Wuhan, China; ^7^ Key Laboratory of Ruminant Bio-products of Ministry of Agriculture and Rural Affairs, Huazhong Agriculture University, Wuhan, China; ^8^ International Research Center for Animal Disease, Ministry of Science and Technology, Huazhong Agricultural University, Wuhan, China

**Keywords:** *Mycoplasma bovis*, ANXA2, adhesion, invasion, RNA-seq, IL-17 signal pathway, CD44 isoforms

## Abstract

*Mycoplasma bovis* (*M. bovis*) is an important pathogen of the bovine respiratory disease complex, invading lower respiratory tracts and causing severe pneumonia. However, its molecular mechanism largely remains unknown. Host annexin A2 (ANXA2) is a calcium-dependent phospholipid-binding protein. The current study sought to determine whether ANXA2 could mediate *M. bovis* adhesion and invasion thereby affecting its induction of inflammatory response. ANXA2 expression was upregulated in *M. bovis*-infected bovine lung epithelial cells (EBL), and blocking ANXA2 with an anti-ANXA2 antibody reduced *M. bovis* adhesion to EBL. Compared with uninfected cells, more ANXA2 was translocated from the cytoplasm to the cell surface after *M. bovis* infection. Furthermore, RNA interference knockdown of ANXA2 expression in EBL cells resulted in a significant decrease in *M. bovis* invasion and F-actin polymerization. Next, the transcriptomic study of *M. bovis*-infected EBL cells with and without ANXA2 knockdown were performed. The data exhibited that ANXA2 knockdown EBL cells had 2487 differentially expressed genes (DEGs), with 1175 upregulated and 1312 downregulated compared to control. According to GO and KEGG analyses, 50 genes potentially linked to inflammatory responses, 23 involved in extracellular matrix (ECM) receptor interaction, and 48 associated with PI3K-AKT signal pathways were upregulated, while 38 mRNA binding genes, 16 mRNA 3′-UTR binding genes, and 34 RNA transport genes were downregulated. Furthermore, 19 genes with various change-folds were selected for qPCR verification, and the results agreed with the RNA-seq findings. Above all, the transcription of two chemokines (IL-8 and CXCL5) and a key bovine β-defensin TAP in IL-17 signaling pathway were significantly increased in ANXA2 knockdown cells. Moreover, ANXA2 knockdown or knockout could increase NF-κB and MAPK phosphorylation activity in response to *M. bovis* infection. Additionally, ANXA2 knockdown also significantly decreased the CD44 transcripts *via* exon V3 and V7 skipping after *M. bovis* infection. We concluded that *M. bovis* borrowed host ANXA2 to mediate its adhesion and invasion thereby negatively regulating molecular expression essential to IL-17 signal pathway. Furthermore, CD44 V3 and V7 isoforms might contribute to this ANXA2 meditated processes in *M. bovis* infected EBL cells. These findings revealed a new understanding of pathogenesis for *M. bovis* infection.

## Introduction

*Mycoplasma bovis* (*M. bovis*) is one of the major causative agents of the bovine respiratory disease complex, which is associated with severe inflammatory reactions such as bronchopneumonia, mastitis, otitis, arthritis, and keratoconjunctivitis (1). This pathogen is widely distributed throughout the world and causes significant economic losses in the dairy and beef cattle industries. *M. bovis* is thought to be spread primarily through direct contact with infected animals and is capable of establishing persistent infections after clinical signs have subsided (2). Studies suggest that the close extracellular association of *M. bovis* with host cells, followed by adhesion and invasion of bovine tracheobronchial epithelial cells, is the most effective way of transmission (3, 4). Several studies have found that in initial cellular interactions, *M. bovis* could bind to ECM, specifically plasminogen and fibronectin (5–7). As described for other bacteria, binding to plasminogen and fibronectin may facilitate invasion and dissemination in the host (8). *M. bovis* has potential to infiltrate a variety of bovine cell types *in vitro*, including primary embryonic calf turbinate cells, embryonic bovine lung (EBL) cells, and embryonic bovine tracheal (EBTr) cells (1, 3, 9). *M. bovis* is also cause of cellular inflammatory responses by increasing the expression of proinflammatory cytokines such as IL-1β, IL-6, IL-8, MMP-1, and MMP-3, leading to the cell and tissue damage (10). In contrast, it inhibits the innate immunity to avoid the clearance, resulting in persistence (11). However, the mechanism by which *M. bovis* regulates the host’s inflammatory response is not fully understood. Understanding this underlined molecular mechanism is critical to elucidating the pathogenicity of *M. bovis*.

The calcium and phospholipid binding protein Annexin A2 (ANXA2) is frequently associated with the cell membrane and cytoskeleton (12). It belongs to the S100 protein family, which is made up of small Ca^2+^-binding proteins with the typical architecture of actin-, mRNA-, and S100A10-binding domains (13). Generally, ANXA2 forms a heterotetrametric complex known as A2t, which is composed of two ANXA2 monomers connected by an S100A10 dimer and found on the inner and outer leaflets of the plasma membrane. Furthermore, the ANXA2 complex has been shown to bind with fibrinolytic system, plasminogen, and tissue plasminogen activator components, resulting in faster activation of the serine protease plasmin and hence enhanced pathogenesis (14). In addition, ANXA2 Tyr phosphorylation is also linked to cell surface translocation to regulates in the dynamics of actin protein (13). Recently, ANXA2 has been shown to be involved in a variety of intracellular functions, including membrane domain organization, vesicular trafficking, and membrane-cytoskeleton contacts, thereby contributing to the bacterial or viral adhesion, entry, and propagation into the host cells (15–18). Furthermore, it is involved in the cell inflammation and host defense (19) *via* ROS, IL-17A (20), or the TRAM-TRIF pathway (21). In mycoplasma, ANXA2 could mediate *M. pneumoniae* community-acquired respiratory distress syndrome (CARDs) toxin by binding to eukaryotic cells and increasing vacuolization (22). ANXA2 also interacts with the N-terminus of *M. hyorhinis* p37 protein and activates its downstream NF-κB pathway mediating the *M. hyorhinis* infection (23). It can also interact with a heat shock protein GroEL of *M. gallisepticum* resulting in infection (24). However, its potential function in *M. bovis* infection has not been clearly documented.

This study sought to investigate the role of AnxA2 during *M. bovis* infection in EBL cells in order to contribute for understanding of *M. bovis* pathogeneses. The results revealed that ANXA2 expression affected the adhesion and invasion of *M. bovis* to EBL and hence likely negatively regulated the inflammatory response by *M. bovis*.

## Materials and methods

### Cells and bacterial culture conditions

The bovine lung epithelial (EBL) cell line was grown in heat inactivated 10% fetal bovine serum supplemented Dulbecco’s modified Eagle medium (DMEM, Gibco, USA) containing at 37°C, 5% CO_2_. *M. bovis* HB0801 (GenBank accession no. NC018077. 1) was grown in a modified PPLO medium (BD Difco™, San Diego, USA) supplemented with 10% heat inactivated horse serum for 36 h at 37°C (Hyclone, South Logan, USA). Mycoplasmas were harvested for infection at the mid-exponential phase of growth after centrifugation for 5 min at 12, 000 rpm to get pellet which was washed three times with PBS and resuspended in DMEM to original volume. Using a standard plate colony counting method, *M. bovis* was quantified as colony-forming units (CFU) per milliliter (25).

### *M. bovis* invasion assay

*M. bovis* invasion assay was determined using a previously described method (26). Briefly, *M. bovis* HB0801 strain was grown to mid-exponential phase, up to 1 × 10^9^ CFU/mL, washed three times with sterile PBS and resuspended in DMEM to original volume. Then *M. bovis* culture was diluted and infected EBL cells to achieve a final multiplicity of infection (MOI) values of 1:10, 1:100, and 1:1000, respectively. After 4, 8, 12, and 24 h post infection (hpi), cells were washed three times and then incubated at standard conditions with 500 μL cell medium containing gentamicin at a final concentration of 400 μg/mL for an additional 3 h to kill the extracellular mycoplasmas. The cells were then washed again, and 1 mL of sterile water was added to lyse the cells to release the intracellular mycoplasma. Finally, the suspension was serially diluted 10-fold, and invaded mycoplasmas were counted using the plate colony counting method. Each treatment was done in triplicate, and the experiments were done three times.

### Analysis of ANXA2 expression during *M. bovis* infection

To see the expression of ANXA2 in EBL cells during *M. bovis* infection, the EBL cells were grown as described above and were seeded (2 ×10^5^ cells/well) in 12 well plates. After overnight growth, the cells were exposed to *M. bovis* at different MOI (1:10, 1:100, 1:1000). The total RNA was extracted from EBL cells using TRIzol reagent following the manufacturer instructions after 0, 4, 8, 12, 24 h post infection of *M. bovis*. Then the cDNA was made using a HiScript 1^st^ Strand cDNA synthesis kit (Vazyme Biotech, China). Next, on an ABI Real-time PCR system, quantitative PCR was performed using SYBR Green master mix (Vazyme Biotech, China). The threshold cycle (Ct) value associated with each target gene’s RNA level was standardized to the level of β-actin expression, and the relative expression was analyzed using the 2^-ΔΔCt^ method previously described (27). **Supplementary Table S1** shows the primer sequences that were used.

The western blot assay was carried out to detect ANXA2 protein expression according to the previously described protocol (27). Briefly, the cells were infected with *M. bovis* at a MOI of 1000 and the protein was extracted at 0, 4, 8, 12, 24 hpi using RIPA buffer containing a protease inhibitor. The proteins were then quantified using BSA kit and separated by 12% SDS-PAGE and transferred to PVDF membranes. The membranes were blocked in TBST (1% Tween-20 in PBS, pH 7.4) with 5% skimmed milk. After three washes with TBST, they were incubated overnight at 4°C with rabbit anti-ANXA2 polyclonal antibody (Abcam, Cambridge, UK) or anti-β-actin monoclonal antibody (Proteintech, Chicago, USA) pre-diluted at 1:1000. After washing, the membranes were incubated with HRP-conjugated goat anti-mouse or anti-rabbit IgG antibody (Southernbiotech, Birmingham, USA), and the protein bands were detected using an enhanced chemiluminescence reagent (Advansta, California, USA). ImageJ software was used to perform the band intensity analysis.

### Effects of anti-ANXA2 antibody on *M. bovis* binding and invasion to EBL cells

EBL cells were seeded in 24-well plates at a density of 2×10^5^ cells. After overnight growth, monolayers were washed with PBS and incubated with mouse anti-ANXA2 monoclonal antibody at different concentration (0.1 μg/ml, 1.5 μg/ml, 3 μg/ml, Santa Cruz Biotechnology, Dallas, USA) or HRP-conjugated goat anti-mouse IgG monoclonal antibody as negative control (3 μg/ml, Millipore, Massachusetts, USA) for 1 h at 4°C according to the literature (28). Then, 2 × 10^8^ CFU/ml *M. bovis* was added and maintained for 30 min. The cells were washed three times with PBS to remove unbound mycoplasmas and lysed by pure sterile water. The suspension was serially diluted and plated on PPLO agar plate to count adhered bacteria. Similarly, the effects of anti-ANXA2 antibody on *M. bovis* invasion to EBL cells was assessed by gentamicin invasion assay at a concentration of 3 μg/ml after 12 h post infection as described above. Each treatment was repeated three time in triplicate.

### Assessment of ANXA2 location during *M. bovis* infection

To see the effects of *M. bovis* on location of ANXA2, the plasma membrane and cytosolic proteins of the mock- and *M. bovis*- infected EBL cells at 12 hpi were isolated using Minute™ plasma membrane protein isolation and cell fractionation kit (Invent Biotechnologies, Plymouth, Minnesota, USA) as manufacturer’s recommendations. The abundance of ANXA2 on the cell surface and in the cytoplasm was tested by the western blot assay as described above. The following appropriate primary antibodies were used for immunodetection: mouse anti-ANXA2 monoclonal antibody (SantaCruz Biotechnology, Dallas, USA), mouse anti-Na^+^/K^+^ ATPase (sigma-Aldrich, St louis, MO, USA) and rabbit anti-tubulin (Abcam, Cambridge, UK). After incubation overnight at 4 °C, the blots were exposed to the appropriated secondary antibody. For indirect immunoflurorescence assay, cells were immunoblotted with rabbit anti-ANXA2 Ab (1:200) and with mouse anti-*M. bovis* 1c11 mAb (1:1000), followed with fluorescein isothiocyanate (FITC)-labled goat anti-mouse IgG (Invitrogen) and Cy3 Red-labeled goat anti-rabbit IgG (Invitrogen) as secondary antibody. DAPI was used to stain the cell nuclei (Beyotime Technology, China). Finally, the slides were covered and examined under a confocal laser fluorescence microscope (Olympus FV1000 and IX81, Tokyo, Japan).

### Knockdown of ANXA2 expression with small interfering RNA and detection of its effect on *M. bovis* invasion

**Supplementary Table S1** lists the specific sequences of small interfering RNA (siRNAs) to the ANXA2 gene (siANXA-1, -2, -3) and control siRNA (siCtrl) synthesized by GenePharma (Shanghai, China). EBL cells were transfected with the optimal siANXA2 or siCtrl using the JetPRIME transfection reagent (Polyplus, NY, USA) per the manufacturer’s instructions. The expression of ANXA2 was measured 48 h after transfection using qPCR and western blotting assay respectively. The transfected cells were then infected with *M. bovis* at an MOI of 1000 for various times (4, 8, 12, and 24 hpi) and then subjected to the gentamicin invasion assay.

To visualize the actin cytoskeleton after siANXA2 transfection for 24 h, the EBL cells were cultured on the coverslip in 12-well plates and infected with *M. bovis* at a MOI of 1000 for the additional 12 h. PBS was used to rinse the slip before it was fixed in 4% paraformaldehyde for 30 min followed by permeabilization in 0.1% Triton X-100 for 10 min. After blocking the cells in 5% BSA for 2 h, the mouse anti-*M. bovis* monoclonal antibody 1C11 was added and cells were incubated overnight at 4°C. After washing three times in PBS, the slips were incubated with FITC-labeled goat-anti-mouse (1:1000, Life Technologies, USA) and rhodamine-labeled phalloidine (1:200, Cytoskeleton, USA) for *M. bovis* and F-actin expression. DAPI was used to stain the cell nuclei (Beyotime Technology). Finally, the slides were covered and examined under a confocal laser fluorescence microscope (Olympus FV1000 and IX81, Tokyo, Japan).

### RNA-seq analysis of infected EBL cells with siANXA2 or siCtrl

The total RNA of *M. bovis-*infected and siANXA2-transfected groups, as well the group infected with *M. bovis* but transfected with siCtrl was extracted using TRIzol reagent (Invitrogen, Thermo Fisher Scientific, USA) and stored at −80°C. When RNA quality testing using an Agilent Bioanalyzer 2200 (Agilent) instrument met the RIN ≥ 9 requirement, samples were sent to the Novogene Company (Beijing, China) for cDNA library construction and RNA-seq as previously described (29).

### Data processing and screening of differentially expressed genes

Sequence reads were filtered for quality, and adapter sequences were trimmed using a custom Perl script that identifies exact string matches to a user-supplied adapter sequence ratio (30). The clean data were mapped to the reference genome to calculate the alignment using HISAT2. Gene expression was represented by fragments per kilobase of exon per million fragments mapped (FPKM). The differential gene transcription analysis was performed using DESeq2 R package (1.16.1) of three biological replicates per condition and selected the genes that had a 1-fold change were further analyzed. The resulting *p*-values were adjusted using the Benjamini and Hochberg’s approach for controlling the false discovery rate. Genes with an adjusted *p*-value <0.05 found by DESeq2 were assigned as differentially expressed as previous reference (31).

### GO and pathway analyses

We used GO analysis to determine the biological implications of our data’s DEGs function. DEGs were uploaded to the database for gene ontology (GO) and the Kyoto Ecyclopedia of genes and genomes (KEGG) analyses, as well as for annotation, visualization, and integrated discovery (DAVID). With a corrected *p <* 0.05, GO terms, and KEGG pathways were considered significantly enriched.

### Identification of changes in gene expression by real-time PCR

In order to validate the results, RNA samples were isolated from siANXA2 and siCtrl. Subsequently, 1μg of total RNA and a reverse transcription kit (Vazyme, Nanjing, China) were used to make cDNA, which was then reverse-transcribed according to the manufacturer’s instructions. SYBR Green master mix was used for qRT-PCR (Vazyme, Nanjing, China). Each target gene’s expression was normalized to that of β-actin. The primer sequences used in this study are shown in **Supplementary Table S1**.

### Generation of the ANXA2-KO EBL cells

ANXA2-KO EBL cells were constructed by using the CRISPR/Cas9 system as described previously (32). The small guide RNA (sgRNA) sequence targeting the bovine ANXA2 gene (5’- GGCCCAAAATCACTGTCTCC -3’) was cloned into lentiCRISPR v2 vector and applied to construct the recombined lentivirus. EBL cells were infected with the ANXA2 lentiCRISPR v2 lentivirus or the empty vector lentiCRISPR v2 lentivirus (negative control). After 48-60 h post infection, puromycin (2.0 mg/ml) was added to select the positive clones. At the end, the monoclonal cells acquired by using the limiting dilution method were expanded and the knockout of ANXA2 was confirmed by western blotting assay.

### Detection of the molecules critical to NF-κB and MAPK signaling pathways

The EBL cells were transfected with siANXA2 or siCtrl and then infected with *M. bovis* at a MOI of 1000. While the ANXA2-KO cells (KO) and EBL wildtype cells (WT) were also infected with *M. bovis* at the same condition. Cells were harvested at 0, 4, 8, 12, and 24 hpi and lysed with RIPA lysis buffer containing a proteinase inhibitor cocktail (Thermo Scientific, Rockford, IL), and protein concentrations were determined by the Bradford assay and equalized before loading. Equal amounts of protein samples were separated on 12% SDS-PAGE, transferred to PVDF membranes, and probed with the following primary antibodies: Rabbit anti-ANXA2 polyclonal antibody, Mouse anti-NF-κB p65 polyclonal antibody, rabbit anti-phospho-NF-κB p65 polyclonal antibody, rabbit anti-ERK polyclonal antibody, rabbit anti-phospho-ERK polyclonal antibody, rabbit anti-JNK polyclonal antibody, rabbit anti-phospho-JNK, rabbit anti-p38 polyclonal antibody and rabbit anti-phospho-p38 polyclonal antibody (Cell Signaling Technology, Massachusetts, USA), then followed by washing and incubation with respective HRP-conjugated goat anti-mouse or anti-rabbit IgG (SouthernBiotech). An enhanced chemiluminescence detection system was used to visualize the reactivity, and the band intensity was analyzed using ImageJ software.

### Identification of changes in alternative splicing

The alternative splicing events were analyzed using replicate multivariate analysis of transcript splicing (rMATS) software (33) to identify siANXA2-induced differential alternative splicing (AS) events. AS events were classified into five basic types, and differences in AS of genes were considered significant with a 5% FDR cutoff.

### Splicing assay

For each sample, the total RNA was erased with a gDNA eraser for 2 min before reverse transcription with an oligo primer (Vazyme, Nanjing, China). The cDNA samples were subjected to qPCR analyses with primers corresponding to the flanking exons of the alternatively spliced exons (**Supplementary Table S1**), and the PCR products were analyzed by TBE gel electrophoresis and quantitated by real-time PCR for exon skipping analyses.

### Statistical analysis

The data are presented as means ± SD (standard deviation) of three biological replicates for each sample. GraphPad Prism software (Version 7.0) was used for statistical analysis and significance was determined using a two-tailed student’s *t*-test for one comparison and ANOVA for multiple comparisons. Differences were considered statistically significant if *p <*0.05 and highly significant if *p* <0.01, *p* <0.001, *p* <0.0001, marked in the figures with *, **, *** and **** respectively.

## Results

### *M. bovis* infection increased the ANXA2 expression in the EBL cells

**Figure 1:**
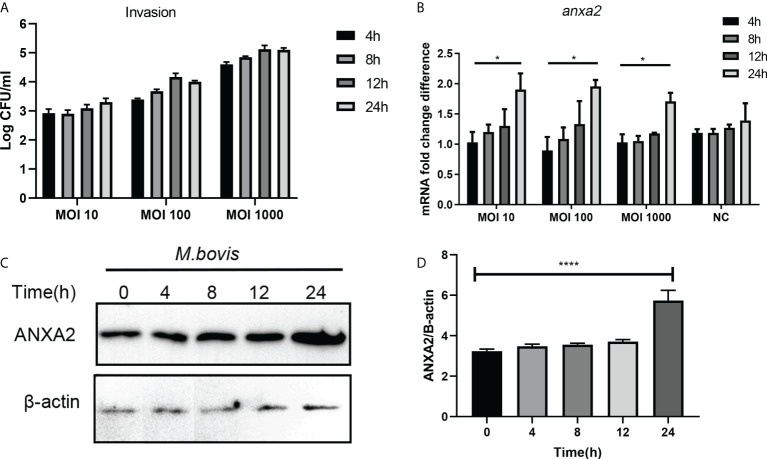
*M. bovis* infection increased the ANXA2 expression in EBL cells. **(A)** The number of invaded *M. bovis* in EBL cells at different infection time points and MOI. **(B)** The mRNA levels of AnxA2 gene in EBL cells infected with *M. bovis* at different MOI and infection time. **(C)** Expected AnxA2 protein bands at different time points of *M. bovis* infection. **(D)** Image J software analyzed band intensity of ANXA2 protein in **(C)**. Data represent means ± SD. *p < 0.05, ****p < 0.0001.

In *M. bovis*-infected EBL cells, the number of invaded bacteria increased as the MOI and infection time increased from 1:10 to 1:1000, and 4 hpi to 24 hpi respectively (**Figure 1A**). *M. bovis* infection increased the expression of ANXA2 gene at mRNA and protein level as indicated by result of qPCR and western blotting data analysis respectively. The mRNA level of ANXA2 gene increased gradually with infection time, and the difference between 4 and 24 hpi became significant (*p* < 0.05) (**Figure 1B**). Similarly, the protein band density significantly increased with infection time (**Figures 1C, D**), indicating that *M. bovis* infection promotes the expression of ANXA2 gene in EBL cells. In addition, we examined the S100A10, the interactive protein of ANXA2, was also upregulated during *M. bovis* infection both at higher and lower MOI at protein level using Western blotting analysis, but not in mock group (**Supplementary Figures S1A, B**).

### ANXA2 is essential for *M. bovis* adhesion to EBL cells through its translocation from cytoplasm to the membrane during infection

**Figure 2:**
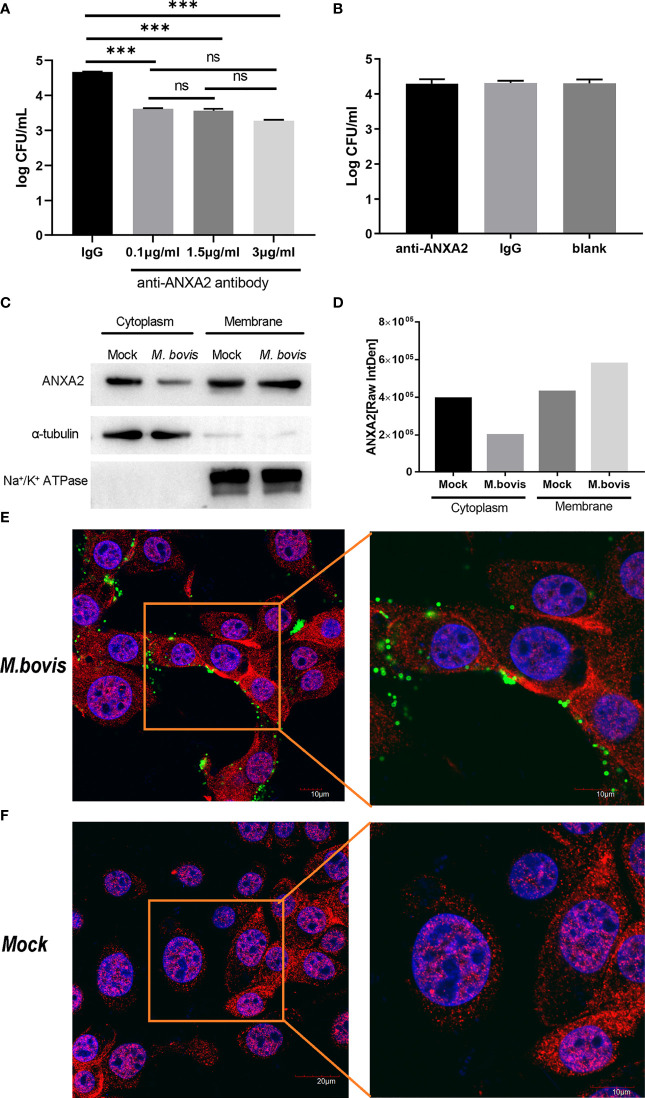
*M. bovis* infection promotes the recruitment of ANXA2 from cytoplasm to membrane surface of EBL cells which in turn is necessary for *M. bovis* adhesion. **(A)** *M. bovis* adhered with EBL cells was reduced by an anti-ANXA2 monoclonal antibody (mAb) in a dose-dependent manner. Data represent means ± SD. ns means no significant, ***p < 0.001. **(B)** Cells treated with an anti-ANXA2 mAb before *M. bovis* infection did not affect *M. bovis* invasion. **(C)**. The levels of ANXA2 in the cytoplasmic and membrane fractions of mock- and *M. bovis*- infected EBL cells at 12 hpi using α-tubulin as a cytoplasmic protein marker and Na^+^/K^+^ ATPase as a membrane protein marker. **(D)** ImageJ was used to analyze the band grey intensity of the western blot assay **(C)**, which was expressed as the raw integrated density (Raw IntDen). **(E**, **F)** Observation of ANXA2 location using confocal microscopy. Live *M. bovis* was infected into EBL cells respectively and set a noninfected group as negative control. After 12 hpi, ANXA2 was detected using rabbit polyclonal anti-ANXA2 and secondary goat anti-rabbit IgG conjugated with Alexa Fluor 555 (red), and *M. bovis* was detected using anti-*M. bovis* monoclonal antibody 1C11 as the primary antibody, followed by a secondary goat anti-mouse IgG antibody conjugated with Alexa Fluor 488 (green), the nuclei were counterstained with DAPI (blue).

The number of *M. bovis* binding to EBL cells was significantly reduced by anti-ANXA2 antibody treatment as compared to control, but there was no significant difference among three different concentrations (**Figure 2A**). However, no significant difference in *M. bovis* invasion was observed between the anti- ANXA2 mAb treated and the negative control treated EBL cells (**Figure 2B**), indicating that the anti-ANXA2 mAb treatment only inhibited the *M. bovis* adhesion. Furthermore, the ANXA2 levels in the cytoplasm and membrane were determined using a western blot assay with α-tubulin as the cytoplasmic protein marker and Na^+^/K^+^ ATPase as the membrane protein marker (**Figure 2C**). ANXA2 levels in the cytoplasm and membrane parts of mock-infected cells were comparable in *M. bovis*. However, in *M. bovis* infected cells, the ANXA2 levels in the membrane were approximately two folds higher than those in the cytoplasm, indicating the occurrence of ANXA2 translocation from the inside to the surface of the cells (**Figure 2D**). Similar results were observed by confocal microscopy showing that, the fluorescence of ANXA2 (red) at the cell surface was stronger in *M. bovis* infected EBL cells than in mock-infected EBL cells (**Figures 2E, F**), suggesting that ANXA2 might affect *M. bovis* binding to the cell on the cell membrane.

### ANXA2 knockdown decreases *M. bovis* invasion and subsequentactin polymerization

**Figure 3:**
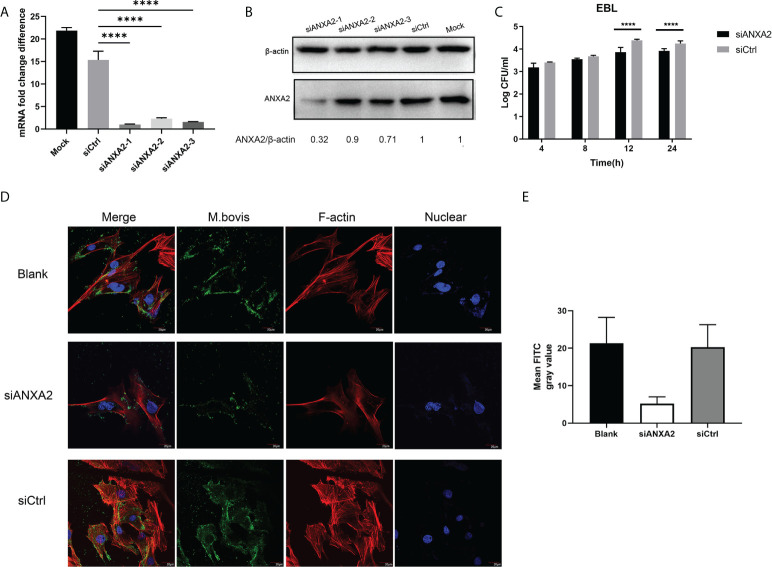
Effects of ANXA2 knockdown with siANXA2 on *M. bovis* invasion and actin polymerization in EBL cells. **A**, **B**. In siRNAs transfected EBL cells, qPCR **(A)** and western blot analysis **(B)** were used to confirm the inhibition of ANXA2 expression by three siANXA2 (siANXA2-1, siANXA2-2, and siANXA2-3) at the mRNA and protein levels. **(C)** Gentamicin invasion assay after ANXA2 knockdown with siANAX2-1 revealed that the amount of intracellular *M. bovis* significantly reduced after 12 and 24 h of infection. The shown data are the mean values of three independent experiments in triplicate. Standard deviations of measurements are indicated by error bars. ****p < 0.0001. **(D)** Confocal laser scanning micrographs of EBL cells without or with ANXA2 knockdown by siANAX2-1 and infected with *M. bovis* at a magnification of 1000 for 12 h. Actin was stained red with rhodamine phalloidin, and the nuclei were counterstained with DAPI (blue), whereas *M. bovis* was stained with anti-*M. bovis* mAb and FITC-labeled secondary antibodies (green). The more actin in the bundle and more *M. bovis* associated with EBL cells was noticed in blank and siCtrl than the cells with ANXA2 knockdown by siANXA2-1. **(E)** Quantitative analysis of FITC channel intensity representing *M. bovis* staining. Ten cells from three different sights were randomly selected for analysis. Values are presented as mean ± SD.

Our qPCR results showed that, the siANXA2-1 has best inhibitory effects on expression of ANXA2 followed by siANXA2-3 and siANXA2-2 (**Figure 3A**). Concomitant to these qPCR results, the western blotting assay also showed the protein level of ANXA2 was maximum inhibited by siANXA2-1 followed by siANXA2-3 and siANXA2-2 (**Figure 3B**) which was then selected for *M. bovis* invasion assay. The number of invaded *M. bovis* in siANXA2-1 transfected EBL cells was significantly (*p* < 0.0001) reduced as compared to siCtrl transfected EBL cells (control) irrespective of infection time point indicating that ANXA2 plays a significant role in the invasion of *M. bovis* into EBL cells (**Figure 3C**).

Furthermore, the confocal microscopy was used to visualize the F-actin bundles and assess the degree of *M. bovis* infection using indirect immunofluorescence assay. The morphology of stained F-actin was vigorously bundled and was similar to the blank and siCtrl-transfected cells although both cells were infected with *M. bovis* (**Figure 3D**). However, F-actin bundles were disassembled in siANXA2-transfected cells, indicating that ANXA2 expression enhances polymerization of F-actin (**Figure 3D**). Quantitative analysis of the mean grey intensity of FITC channel from the images showed that, nearly 2-fold less *M. bovis* labeled with FITC in the siANXA2 transfected cells was existed than in siCtrl and blank group (**Figure 3E**).

**Figure 4:**
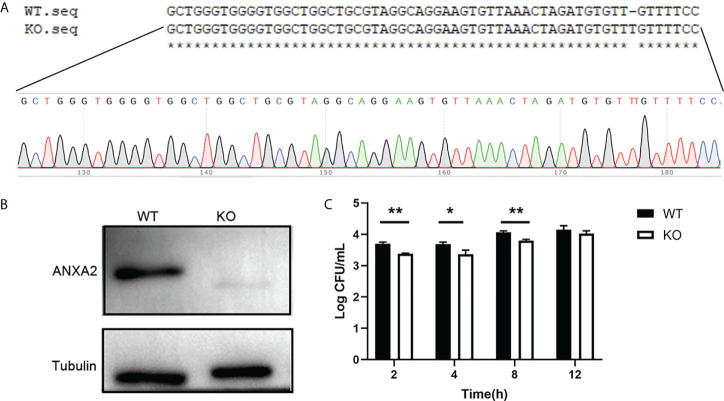
Generation of ANXA2-KO EBL cells by using CRISPR/Cas9 system. **(A)** BLAST analysis of sequences around the PAM sequence of WT EBL and ANXA2-KO EBL cell lines. **(B)** ANXA2 knockout was confirmed by western blotting with an anti-ANXA2 mouse antibody. **(C)** The invasion ability of *M. bovis* in ANXA2-KO EBL cells. ANXA2-KO EBL cells (KO) or wild-type EBL cells (WT) were infected with *M. bovis* at an MOI of 1000. The intracellular bacteria were determined using gentamicin invasion assay (mean ± S.D., * *p*<0.05, ** *p*<0.01).

In addition, the ANXA2-KO EBL cells were produced and confirmed by western blotting (**Figures 4A, B**) to determine the effect of ANXA2 on *M. bovis* invasion. Results showed that intracellular *M. bovis* were significantly decreased in the ANXA2-KO EBL cells compared with those in the WT EBL cells at 2, 4, 8 hpi (**Figure 4C**), indicating that ANXA2 does play a critical role in *M. bovis* infection.

### RNA-seq analysis identified the differentially expressed genes in *M. bovis-* infected siANXA2 and siCtrl EBL cells

**Figure 5:**
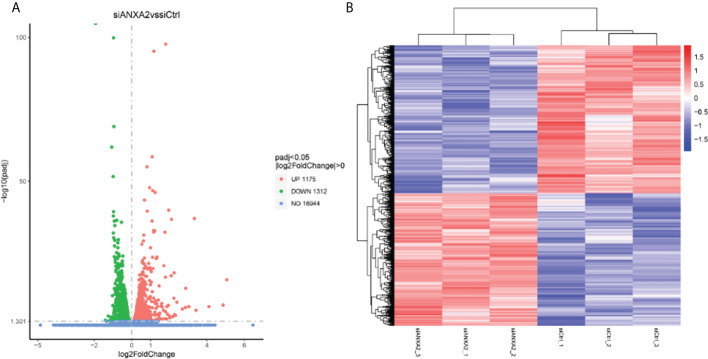
Identification of differentially expressed genes (DEGs) by RNA-seq data analysis between the *M. bovis* infected-siANXA2 and siCtrl EBL cells. **(A)** Volcano plots of DEGs expressed between the siANXA2 group and siCtrl group. The blue, red and green dots represent the genes that were not differentially expressed (*p* > 0.05), significantly upregulated, and significantly downregulated (*p* < 0.05), respectively. **(B)** Heatmap of the DEGs differentially expressed between the siANXA2 group and siCtrl group. Blue-to-red scale indicates low to high DEGs expression levels. The log_2_(FPKM) value is indicated by the gradient color barcode at the right top. Each row corresponds to a gene, and each column corresponds to a sample.

To determine the further role of ANXA2 in the infection process, we next performed an RNA-seq analysis for *M. bovis* infected- siANXA2 transfected EBL cells. Among the 16,944 genes tested, 2,487 were significantly modulated in siANXA2-transfected compared to control (siCtrl-transfected) cells after *M. bovis* infection (**Supplementary Table S2**). Further investigation revealed that 1175 genes were significantly upregulated (**Supplementary Table S3**), while 1312 genes were significantly downregulated (**Figure 5A** and **Supplementary Table S4**). As shown in the heatmap, each row represents the expression level of each gene in different samples, and each column represents the expression level of all genes in each sample (**Figure 5B**).

**Figure 6:**
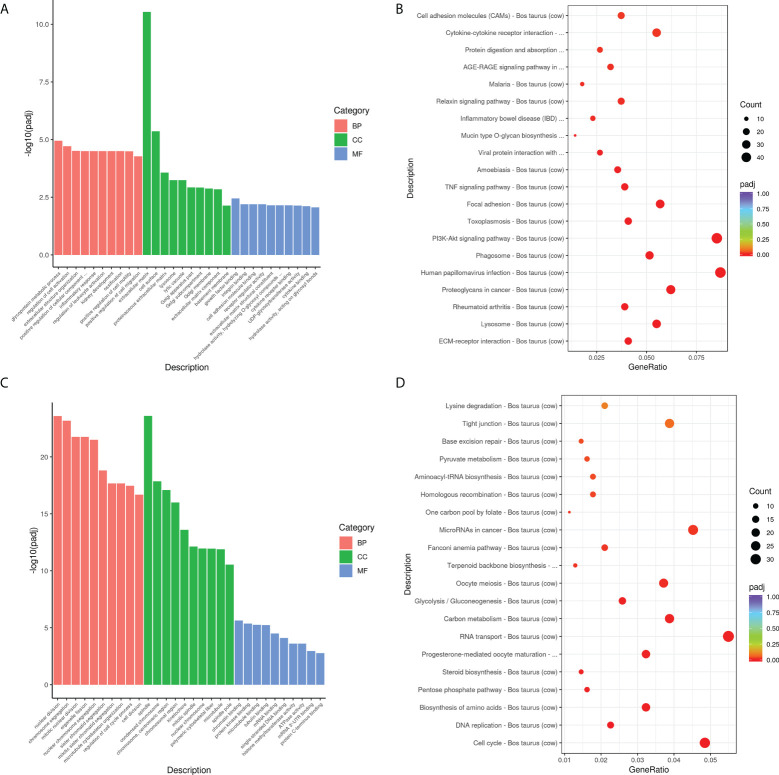
Biological function analysis of DEGs using GO and KEGG enrichment analysis. **(A)** The top 10 GO terms enriched for the upregulated DEGs. The Y-axis represents the number of -log10(padj), and the X-axis represents GO terms. All GO terms are classified into three ontologies: red denotes a biological process (BP), green denotes a cellular component (CC), and blue denotes a molecular function (MF). **(B)** The top 30 KEGG pathways that are enriched in the upregulated genes. The *vertical axis* represents the pathway name, the *horizontal axis* represents the Gene ratio, the size of the dots indicates the number of candidate target genes in this pathway, and the color of the dots corresponds to the different *padj*. **(C)** The top 10 GO terms enriched for the downregulated DEGs. **(D)** The top 30 KEGG pathways enriched in the downregulated genes.

GO and KEGG analyses were used to further investigate the roles of these DEGs in *M. bovis* infected- siANXA2 transfected EBL cells. Among the upregulated DEGs (**Figure 6A** and **Supplementary Table S5**), 30 GO terms were enriched related to *biological processes* such as glycoprotein metabolic process, cell activation regulation, extracellular structure organization, positive regulation of cellular component, and inflammatory response. Extracellular matrix and cell surfaces were the most affected cellular component functions, and affected molecular function was associated with growth factor binding, integrin binding, and cell adhesion molecular activity. Furthermore, KEGG pathway terms revealed that these genes were associated with a variety of pathways, including ECM-receptor interaction, lysosome, rheumatoid arthritis, phagosome, and the PI3K-AKT signaling pathway (**Figure 6B**, fold-change >1.5, *p* < 0.05, **Supplementary Table S6**).

Overall, the top up-regulated genes included those that govern inflammation (IL-6, IL-6R, and TGF), chemotaxis (CXCL5 and CCL5), extracellular matrix protein (ECM1), antimicrobial defensin (Tracheal antimicrobial peptide, TAP), and tissue remodeling (MMP2, MMP9, MMP13). In contrast, the top 30 GO terms in the downregulated DEGs are shown in **Figure 6C** and **Supplementary Table S5**. These were mostly related to nuclear division, chromosome segregation, spindle, and polymeric cytoskeletal fiber. Notably, GO analysis of the downregulated genes revealed that genes which regulate the cell-binding, such as microtubule, tubulin binding, mRNA, and mRNA 3’UTR binding. Additionally, KEGG-term results revealed that the downregulated genes are involved in a variety of pathways, including the cell cycle, DNA replication, amino acid biosynthesis, the pentose phosphate pathway, steroid biosynthesis, and RNA transport (**Figure 6D** and **Supplementary Table S6**).

### Real-time RT-PCR validation of selected DEGs

**Figure 7:**
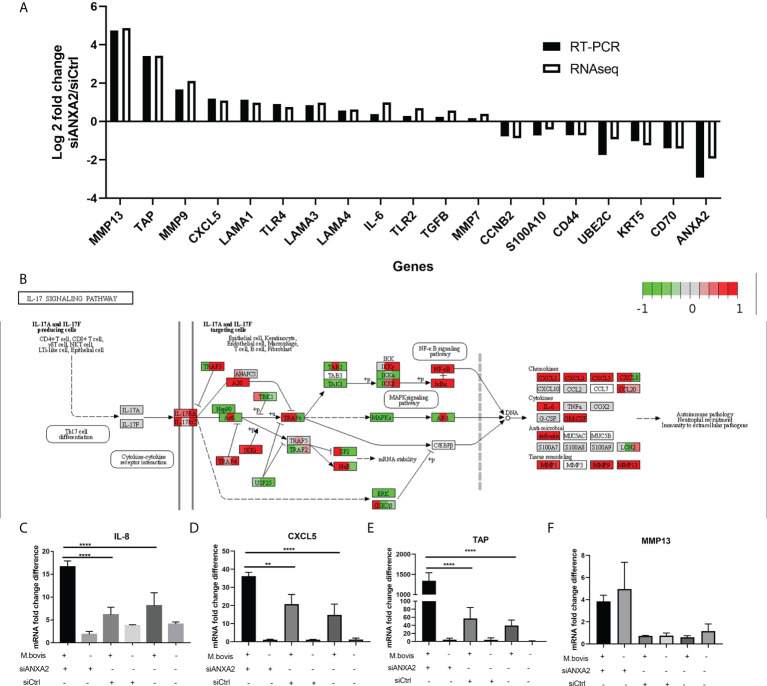
Validation of expression patterns of selected genes using qPCR. **(A)** Log2 Fold changes of mRNA expression levels of 19 DEGs encoding inflammation-related chemokines and cytokines, as well as other randomly selected DEGs by qPCR in *M. bovis* infected EBL cells with or without siANXA2 transfection. **(B)** Validation of DEGs associated with the IL-17 signal pathway in *M. bovis*-infected EBL cells treated with siANXA2 and siCtrl. The DEGs were mapped to IL-17 signaling pathway. The filled red and green color represents upregulated and downregulated genes respectively. **(C–F)**. qRT-PCR validation of DEGs IL-8 **(C)**, CXCL5**(D)**, TAP**(E)** and MMP13 **(F)** genes mapped in the IL-17 signaling pathway in *M. bovis* infected or uninfected cells treated with siANXA2 and siCtrl. The values shown in the graphs are the mean ± SD of three independent experiments. ** represents *p* < 0.01, and **** represents *p* < 0.0001.

As DEGs were primarily enriched in GO functional terms of inflammatory response and extracellular matrix, as well as pathways of ECM-receptor interaction, lysosome, and PI3K-AKT signaling, we chose 19 related DEGs to confirm their expression at the mRNA level and the data reliability obtained by RNA-seq results by qPCR in *M. bovis* infected or mock-infected cells treated with siANXA2 and siCtrl. The mRNA levels of all these selected genes were found to be consistent with the RNA-seq findings, including the downregulated ANXA2 by siANXA2 (**Figure 7A**). There was a significant increase in mRNA levels of MMP13, TAP, MMP9, CXCL5, and IL-6 in siANXA2-transfected group compared to the siCtrl group.

In addition, the upregulated genes (MMP13, TAP, MMP9, CXCL5, and IL-8) were involved in IL-17 signaling pathway after ANXA2 knockdown (**Figure 7B**), implying that ANXA2 may negatively mediate the IL-17 signaling pathway. Furthermore, in *M. bovis*-infected EBL cells treated with siANXA2, the mRNA expression of IL-8 and CXCL5 genes increased by 3-4 folds (**Figures 7C,D**), while TAP expression increased by 20-folds compared to control (*M. bovis* infected-siCtrl-treated EBL cells) (**Figure 7E**). MMP13 did not differ before and after *M. bovis* infection, even though its gene expression increased in ANXA2 knockdown cells compared to siCtrl and blank cells (**Figure 7F**).

### ANXA2 suppression results in upregulation of key molecules expression involved in NF-κB and MAPK signal pathway triggered by *M. bovis*

**Figure 8:**
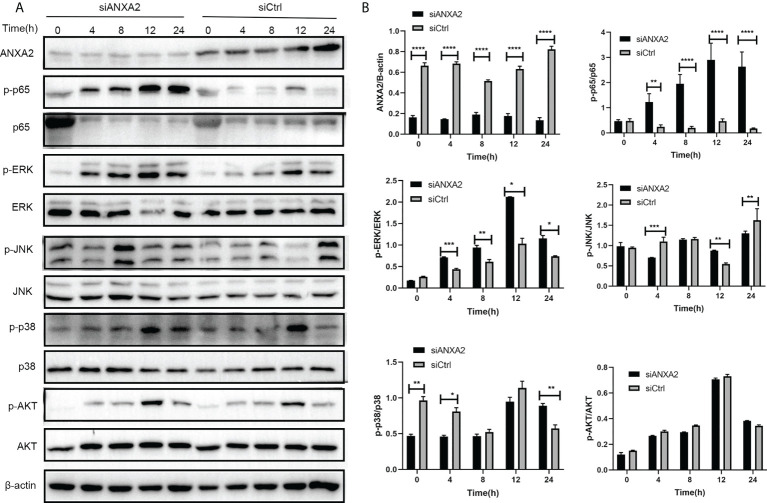
Knockdown of ANXA2 exacerbates the activation of NF-κB and MAPK signaling induced by *M. bovis*. **(A)** EBL cells were transfected with siANXA2 or siCtrl for 24h, then infected with *M. bovis* for the indicated time points. ANXA2 and unphosphorylated and phosphorylated signaling molecules i.e., NF-κB p65, MAPK (ERK, JNK, p38), and AKT were detected in lysates of EBL cells stimulated with *M. bovis* by employing β-actin as an internal reference protein. **(B)** ImageJ software was used to analyze the band intensity of panel A, and the ratio of phosphorylated proteins to unphosphorylated proteins was calculated. The data from three independent experiments were presented as mean values and standard deviations. * from three independent experiments. *p<0.05, **p<0.01, ***p<0.001, ****p<0.0001.

EBL cells were transfected with siANXA2 for 24 h before being stimulated with *M. bovis* at the indicated time intervals. To determine the underlying mechanisms of ANXA2 in inflammatory responses, immunoblotting was used to detect proteins associated with NF-κB and MAPK pathways. We noticed that ANXA2 protein expression was significantly reduced in siANXA2 transfected EBL cells, despite being infected with *M. bovis*. Immunoblotting results also revealed that *M. bovis* infection could significantly increase the phosphorylation of NF-κB p65 and ERK in ANXA2-depleted EBL cells, but has no effect on the phosphorylation of JNK and p38 (**Figures 8A, B**).

**Figure 9:**
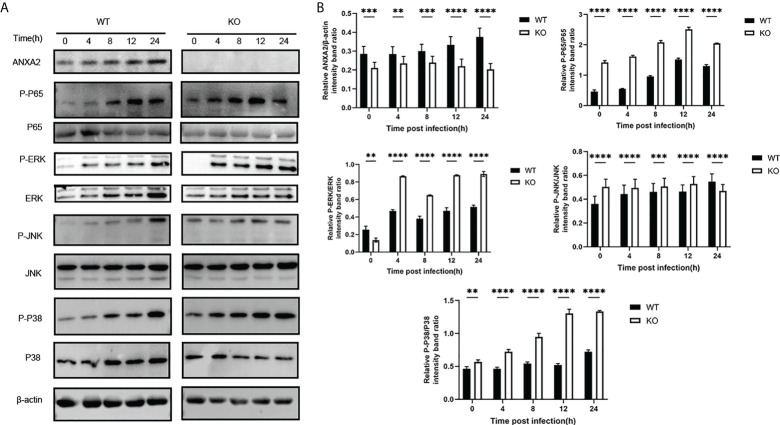
Knockout of ANXA2 exacerbates on the NF-κB and MAPK signaling pathways during *M. bovis* infection process. **(A)** ANXA2-KO EBL cells (KO) or wild-type EBL cells (WT) were infected with *M. bovis* for the indicated time points, and western blotting assay was performed to determine the protein levels of ANXA2 and unphosphorylated and phosphorylated signaling molecules. **(B)** The band intensities were quantified with ImagJ, and the relative ANXA2, phosphorylated-P65, phosphorylated-P38, phosphorylated-JNK levels are shown. The data from three independent experiments are presented as mean values and standard deviations. **p<0.01, ***p<0.001, **** *p*<0.0001.

In addition, based on a ANXA2-KO EBL cell line, we found that the activation of phosphorylated p65 in NF-kB signal pathway and ERK/P38 in MAPK signal pathway was also enhanced during *M. bovis* infection (**Figures 9A, B**). This is supporting evidence to verify the effect of ANXA2 on the activation of NF-κB and MAPK signal pathway. The AKT and phosphorylated AKT weren’t tested here because the previous siANXA2 transfection did not affect their expression.

### ANXA2 knockdown induces alternative splicing events after *M. bovis* infection

The RNA sequencing data were analyzed with rMATS software. Compared to *M. bovis*-infected-siCtrl transfected EBL cells, the *M. bovis*-infected- siANXA2 transfected EBL cells showed 1254 significant alternative splicing events upon knockdown with a 5% FDR cutoff. Interestingly, primary exon skipping (ES) events account for 66. 2% of the differential alternative splicing events. Of these, 47 intron retention and 795 ES events were found to be significantly expressed (**Table 1** and **Supplementary Table S7**).

**Table 1:** Differential alternative splicing events between *M. bovis*-infected EBL cells with siANXA2 and siCtrl.

	Exon skipped	Intron retained	Mutual exclusive exon	Alternative 5′splicing sites	Alternative 3′splicing sites
**Number of total alternative splicing events (genes)**	25893(8508)	705(624)	4239(2478)	296(264)	320(281)
**Number of significant alternative splicing events (genes)**	795(674)	47(47)	346(311)	34(33)	32(32)
**Number of differential alternative splicing events (upregulation/downregulation)**	151(72/79)	7(2/5)	58(34/24)	8(4/4)	4(3/1)
**Percentage of total differential alternative splicing event (%)**	66.2	3.0	25.4	3.5	1.8

### ANXA2 knockdown led to CD44 variant exon skipping after *M. bovis* infection

**Figure 10:**
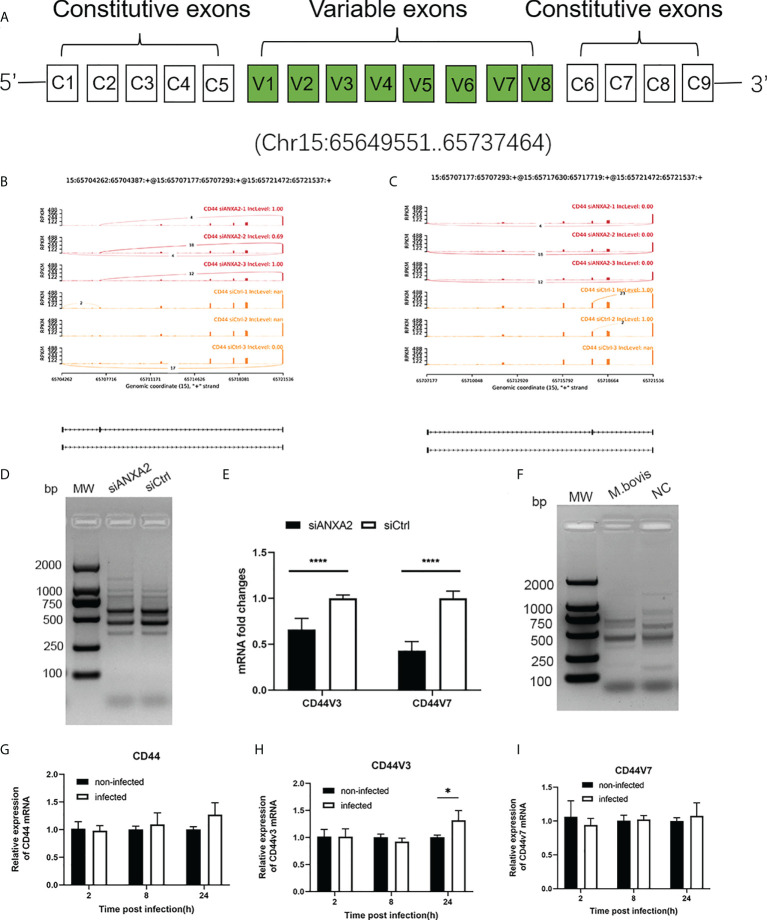
ANXA2 knockdown resulted in alternative splicing of *CD44* after *M. bovis* infection. **(A)**. *CD44* standard and variant exons are depicted schematically, with invariant exons 1-5 and the last five exons and variant exons v1-v7. **B**, **C**. Sashimi plots depicting the region tested for the presence of alternative splicing isoforms due to the skipped exons in *CD44* gene. Genomic coordinates, chromosome number, strand and exon number are indicated below. Inclusion level (IncLevel) represents the ratio of the expression of exon inclusion isoform in the total exon inclusion and exon skipping isoforms, which could be directly show the expression differences of variable splicing events in the siANXA2 and siCtrl group. **D** The inclusion of CD44 exons in alternative splicing in *M. bovis* infected-ANXA2 knockdown cells was validated using RT-PCR. The forward primer was designed on exon C5 of CD44 mRNA, while the reverse primer on exon C6. **E** The differential expression of CD44 v3 and v7 in infected EBL cells was quantified using qPCR between *M. bovis* infected-siANXA2 transfected and control EBL cells (*M. bovis* infected-siCtrl transfected EBL cells. The qPCR results are the mean of three biological replicates. ****p < 0.0001.** F** Semi-quantitative RT-PCR of CD44 isoforms after *M. bovis* infection in EBL cells. **G**-**I** Quantitative PCR analysis of changes in CD44, CD44V3, CD44V7 after *M. bovis* infected at different times. Data represent means ± SD. * *p*<0.05.

Among the DEGs due to ANXA2 silencing in *M. bovis* infected EBL cells, the *CD44* gene was found to experience mostly significant splicing changes. The pre-mRNA of *CD44* gene contains 17 exons, of which the first five and last four were retained in the mature *CD44* mRNA to form the CD44 standard form (CD44S) (**Figure 10A**). The inclusion of some of the rest eight variable exons results in the variant isoforms of CD44. Exon skipping was discovered in *CD44* at exons 3 and 7. The exon 3 from chr15:65717629 nt to 65717719 nt and exon 7 from chr15: 65707176 nt to 65707293 nt were skipped (**Figures 10B, C**). Further, using RT-PCR with forward primers designed on exon C5 and reverse primers designed on exon C6, it was confirmed that ANXA2 knockdown resulted in increased expression of CD44 variant isoforms as evidenced by the presence of more bands over 1 kb in cells with siANXA2 than siCtrl after *M. bovis* infection (**Figure 10D**). However, the mRNA expression of CD44 variant transcripts CD44v3 and CD44v7 were significantly decreased in *M. bovis* infected EBL cells with ANXA2 knockdown (**Figure 10E**). siANXA2 treatment group had more ES spliced transcript events of CD44V3 comparing to siCtrl group, which may explain the downregulation of CD44 mRNA in siANXA2 group. The fragments of CD44 splicing isoforms using RT-PCR with forward primers designed on exon C5 and reverse primers designed on exon C6 were detected and demonstrated that *M. bovis* infection resulted in decreased expression of CD44 variant isoforms as shown in **Figure 10F**. Furthermore, the *in vitro* studies revealed that the mRNA level of CD44v3 was increased, but CD44 and CD44v7 was constant (**Figures 10G, H, I**).

## Discussion

*M. bovis* causes chronic pneumonia, mastitis, and arthritis in cattle, as well as a severe inflammatory response in the lung, mammary gland, and joints. Its infection involves complex and redundant pathways that disrupt the host immune response for persistence and dissemination. The precise molecular mechanisms of *M. bovis* pathogenesis remains unknown and need to be investigated. In the current study, we discovered that *M. bovis* infection in EBL cells enhances the ANXA2 expression as well as its translocation from cytoplasm to the plasma membrane is required for *M. bovis* binding and invasion that are the initial steps in bacteria pathogenesis. Through transcriptome analysis, we discovered extensive information on differential gene expression and alternative splicing in association with ANXA2 suppression. Several key genes are differentially expressed between siANXA2 and siCtrl such as IL-8, CXCL5, and TAP. This process negatively regulates NF-κB and MAPK phosphorylated activity, presumably to control infection with *M. bovis*.

### ANXA2 mediated *M. bovis* infection

ANXA2 exists as a monomer in the cytosol or in a complex with S100A10 to form the ANXA2/S100A10 heterotetramer (A2t) found in the plasma membrane. Both ANXA2 and A2t are involved in a wide range of cellular functions, including endocytosis, exocytosis, membrane domain organization, and membrane-cytoskeleton contacts, *via* two domains: a variable N-terminal domain that interacts with different host proteins, bacteria, and virus proteins and a C-terminal conserved protein core that interacts with Ca^2+^ and phospholipid binding sites (34). First, we demonstrated that ANXA2 mRNA and protein levels increased during the invasion of *M. bovis*, and S100A10, the interacting protein of ANXA2, was also upregulated in *M. bovis* infection, indicating ANXA2 might together with S100A10 participate in its infection. Increased ANXA2 expression has been observed in a variety of cancers (35), viral infections (18), and bacterial invasion processes such as *Escherichia coli* (36) and *Cryptococcus neoformans* (27). Previous research has discovered that ANXA2 contributes to *M. hyorhinis* infection by interacting with p37 at their N-terminal domain (23). ANXA2 is also an important receptor that allows plasminogen (Plg) and tPA to assemble host fibrinolytic machinery on the host cell surface and plays a critical effector role in bacterial adhesion and invasion (28). It was reported that some *M. bovis* adhesins like Enolase (6), FBA (37) could bind to Plg that participates in *M. bovis* adhesion to the host cells.

Our results showed that an anti-ANXA2 antibody reduced the *M. bovis* adhesion to the EBL surface, but there was no significant difference in invaded *M. bovis* after anti-ANXA2 antibody treatment compared to the control, indicating that the surface ANXA2 is ineffective in preventing entry of *M. bovis*. After that, we revealed that *M. bovis* exposure may cause cytoplasmic ANXA2 translocation to the epithelial cell membrane, aiding its adhesion and invasion. As a result, we hypothesize that its function is related to its intracellular distribution and post-translational modification. Previous literature suggests that bovine AnxA2 is subjected to complex regulation by post-translational modifications affecting its cellular functions, including a total of 23 phosphorylation sites where Ser11, Ser25 and Tyr23 representing important phosphorylation sites. It was reported that Ser11 phosphorylation as a regulator of S100A10 binding was involved in fibrinolysis, hemostasis and ion channels. Ser25 phosphorylation was the binding site for mRNA and G-actin. While Tyr23 phosphorylation could decrease the affinity of ANXA2 for liposomes and also impact on the process of endocytosis (13). However, whether these phosphorylation effect on *M. bovis* binding and invasion still need to be confirmed in the further study.

### ANXA2 knockdown decrease *M. bovis* invasion affecting F-actin polymerization and binding to its possible ligand S100A10 and CD44

Bovine ANXA2 contains four highly conserved “annexin core structure” domains that exhibit the classical properties of calcium-dependent lipid binding, RNA binding, S100A10, and F-actin binding sites (13). In our study, although anti-ANXA2 antibody was not enough to block the invasion process, the number of invaded *M. bovis* to ANXA2 knockdown- and knockout- EBL cells was significantly reduced compared to siCtrl transfected or wild type EBL cells, suggesting the surface ANXA2 was not prevent *M. bovis* invasion but cytoplasmic ANXA2 might be play a significant role in the invasion of *M. bovis* into EBL cells. In addition, F-actin disassembled was also caused by the suppression of ANXA2. Since it was reported that actin is a bacteria receptor in *M. hyopneumoniae* (38), we speculated that the actin cytoskeleton was disorder due to ANXA2 silencing possibly influencing *M. bovis* recognition and invasion.

When AXNA2 as a stable heterotetramer composed of two molecules each of ANXA2 and S100A10, it has a higher affinity for membranes found in the subplasmalemmal region (39). *M. bovis* infection caused ANXA2 to translocate from the cytoplasm to the membrane, implying that ANXA2 is dependent on its partner S100A10 to promote *M. bovis* invasion and internalization. S100A10 mRNA expression was also found to be lower in both RNA-seq and RT-PCR analyses after ANXA2 knockdown. Previous research found that S100A10 was required for ANXA2 to mediate transcytosis of *Cryptococcus neoformans* across the endothelium of the brain (27). We hypothesized that ANXA2 knockdown decreased F-actin polymerization and S100A10 expression, implying that ANXA2 may be an indirect contributor to cytoskeleton morphology and thus regulating *M. bovis* invasion into epithelial cells, though this hypothesis needs to be confirmed further.

In addition, ANXA2 is also known as a lipid raft-associated scaffold protein that is localized in the membrane (40). CD44 is the major cell surface receptor for hyaluronic acid and an important component of lipid rafts, which have been shown to interact with ANXA2 in a cholesterol-dependent manner (41). In our study, AXNA2 knockdown reduced the CD44 mRNA levels and induced more AS of CD44 *via* ES, resulting in a greater abundance of CD44 variant isoforms. CD44 AS events are frequently linked to cancer and microbes infection (42, 43). Interestingly, in *M. bovis* infected EBL cells, the various isoforms of CD44 using RT-PCR with forward primers designed on exon C5 and reverse primers C6 was decreased with the mock group (**Figure 10F**). Then the variant isoforms were further detected and the mRNA level of CD44V3 was increased, but CD44 and CD44v7 was constant (**Figures 10G–I**). Although the role of this increased spliced isoform CD44V3 in *M. bovis* infection needs to be confirmed in further study, it could contribute to the adhesion of *M. bovis* and subsequent induction of inflammatory response since it serves as a bridge molecule in adhesion, and migration of lymphocytes and activation of T cells, and the correlation between CD44 isoforms, ANXA2 and *M. bovis* infection is a new interesting finding and might be involved in some important mechanism contributing to *M. bovis* infection.

### Interaction between *M. bovis* and ANXA2 inhibited inflammatory response through specific signaling pathways

Recently, ANXA2 has been shown to contribute to inflammation and host defense and promote the adhesion, entry, and propagation of bacteria or viruses into host cells (19). In this study, we discovered that ANXA2 knockdown results in upregulation of DEGs that were abundant in various pathways such as ECM-receptor interaction, lysosome, rheumatoid arthritis, phagosome, and PI3K-AKT signaling pathway. Several innate immune-related genes, including MMP13, MMP9, CXCL5, TLR4, TLR2, TAP, IL-6, and IL-8, were significantly upregulated. We also show that the production of inflammatory cytokines (such as IL-8, CXCL5, TAP) in IL-17 signaling pathway were significantly increased after ANXA2 knockdown upon *M. bovis* infection.

Experimental evidence shows that ANXA2 is critical for down-regulation of inflammatory events (44). While Zhang et al. in 2015 also found that ANXA2 may be a negative regulator of bacteria-triggered inflammatory responses, as ANXA2 gene depletion exacerbate LPS induced inflammation through its negative regulatory role in the process of P65 nuclear translocation of the NF-κB signaling pathway through TLR4 (21), which is similar to what we observed with ANXA2. In addition, ANXA2 expression promoted a permissive environment for infection, induced suppression of immune activation and reduced the Th-1 cytokine production *in vitro*. ANXA2 possesses redox sensitive cysteines, thus deletion of ANXA2 results in elevated ROS upon oxidative stress, increased activation of ROS-induced pro-apoptotic kinases (such as JNK, P38) (45). Furthermore, *M. bovis* infection relies on MAPK and NF-κB pathways (46, 47). Therefore, we speculate that ANXA2 could negative regulate the two pathways and play a crucial role in *M. bovis* innate immune evasion. These include the potential interaction and modulation of proteins related to IL-17A and its receptor, IL-17RA, as well as the activation of anti-inflammatory proteins by ANXA2 that serves as negative feedback control of IL-17A/IL-17RA mediated signaling. Previous research has found that in ANXA2 KO mice, faster bacterial growth and more severe tissue injury could induce a strong inflammatory response *via* an ANXA2-ROS-IL-17 axis during polymicrobial sepsis (20). While these findings are intriguing and open to further interpretation, several unique ANXA2 actions remain unknown. We also examined the changes in downregulated DEGs using RNA sequencing and discovered that these DEGs were enriched in microtubule, tubulin binding, mRNA, and mRNA 3′UTR binding, as well as the effect on RNA transport. As a result, ANXA2 deficiency may impair actin and RNA binding ability. This study has some limitation. The phenotypes of *M. bovis* infected cells mediated by ANXA2 should be validated with ANXA2 ectopic expression and in different cell types.

In conclusion, our findings suggest that ANXA2 plays an important role in enhancement of *M. bovis* adhesion and invasion by its translocation from the cytoplasm to the cell surface and effect on F-actin polymerization and thereby, negatively regulating *M. bovis*-induced molecule expressions essential to IL-17 signal pathway including TAP, CXCL5, and IL-8. Furthermore, the differential expression of CD44 isoforms is regulated ANXA2 during *M. bovis* infection.

## Data availability statement

The data presented in the study are deposited in the NCBI BioProject repository, accession number PRJNA766044.

## Author contributions

Conceptualization, HZ. Methodology, HZ, DL YZ. Writing original draft preparation, HZ. Writing, review and editing, AG and AR. Software, GZ. Validation, HZ and GZ. Formal analysis, YC, XC, and CH. Supervision, HC, LY and AG. Project administration, AG. Funding acquisition, AG and HZ. All authors have read and agreed to the published version of the manuscript.

## Funding

This work was supported by the Youth Program of National Natural Science Foundation of China (No. 32002290), Youth Program of Natural Science Foundation of Hebei province of China (No. C2018402137), the earmarked fund for China Agriculture Research System of MOF and MARA (Beef/yaks) (No. CARS-37).

## Conflict of interest

The authors declare that the research was conducted in the absence of any commercial or financial relationships that could be construed as a potential conflict of interest.

## Publisher’s note

All claims expressed in this article are solely those of the authors and do not necessarily represent those of their affiliated organizations, or those of the publisher, the editors and the reviewers. Any product that may be evaluated in this article, or claim that may be made by its manufacturer, is not guaranteed or endorsed by the publisher.
