# Supplementary material for: Annexin A2 regulates Mycoplasma bovis adhesion and invasion to embryo bovine lung cells affecting molecular expression essential to inflammatory response
Source: Front Immunol. 2022 Sep 8;13:974006. doi: 10.3389/fimmu.2022.974006 (PMC9493479; doi:10.3389/fimmu.2022.974006)
Supplement: Supplementary file 1 [file DataSheet_1.zip › supplementary tables/Fig.S1 legend.docx]

**Fig.S1** The expression of S100A10 was upregulated during *M. bovis* infection. A. Western blot assay determined the expression of S100A10 upon *M. bovis* infection at two MOI and four infection time points. β-actin was the loading control. B. Then band intensity of the western blot assay was investigated. Standard deviations of measurements are indicated by vertical bars. * *p* < 0.05, ** *p* < 0.01, **** *p* < 0.0001.
