# Supplementary figures and images for: Annexin A2 regulates Mycoplasma bovis adhesion and invasion to embryo bovine lung cells affecting molecular expression essential to inflammatory response
Source: Front Immunol. 2022 Sep 8;13:974006. doi: 10.3389/fimmu.2022.974006 (PMC9493479; doi:10.3389/fimmu.2022.974006)

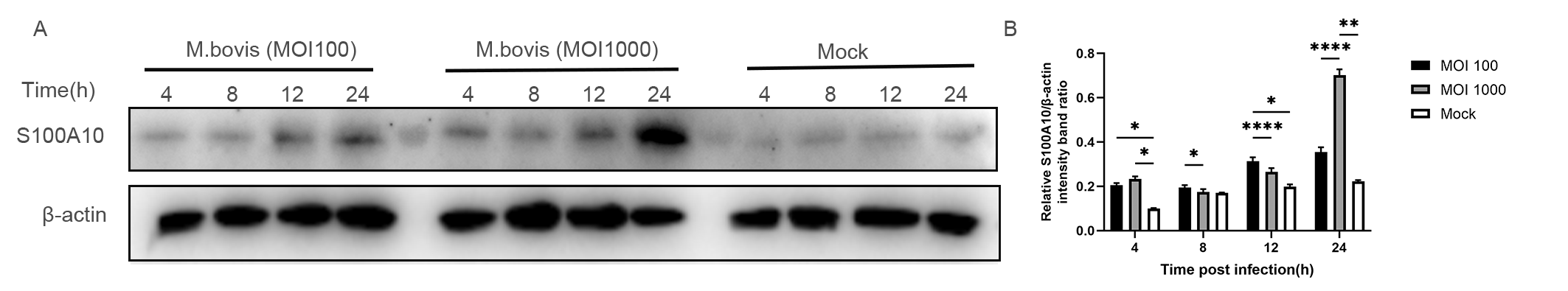

Supplement: Supplementary file 1 [file DataSheet_1.zip › supplementary tables/Fig.S1.tif]
